# Seroprevalence and Risk Factors Associated With Brucellosis in Goats in Nyagatare District, Rwanda

**DOI:** 10.1155/vmi/3400402

**Published:** 2025-08-24

**Authors:** Jean Paul Habimana, Jean Bosco Ntivuguruzwa, Aime lambert Uwimana, Marie Aurore Ugirabe, Eric Gasana, Henriette van Heerden

**Affiliations:** ^1^Department of Veterinary Tropical Diseases, University of Pretoria, Pretoria, South Africa; ^2^School of Veterinary Medicine, University of Rwanda, Nyagatare, Rwanda; ^3^Department of Mathematics, University of Rwanda, Kigali, Rwanda; ^4^Department of Applied Statistics, University of Rwanda, Kigali, Rwanda

**Keywords:** brucellosis, risk factors, Rwanda, seroprevalence

## Abstract

Given the endemic nature of bovine brucellosis in Rwanda, caprine brucellosis, primarily caused by *Brucella melitensis* in goats, may also be prevalent. However, no data exist on the disease's prevalence and associated risk factors in goats, particularly in Nyagatare district. A cross-sectional study was therefore conducted to determine the seroprevalence of brucellosis and to identify herd-level risk factors associated with the disease among goat herds (*n* = 102) across six sectors of Nyagatare district. Serum samples from 612 goats were tested using both the indirect enzyme-linked immunosorbent assay (i-ELISA) and the Rose Bengal Test (RBT), applied in parallel. A systematic questionnaire, pretested for reliability, was used to gather data on potential risk factors for caprine brucellosis. The study found a brucellosis true adjusted seroprevalence of 6.08% and 10.7% using RBT and i-ELISA, respectively. When combining the results from both tests, the overall seroprevalence was 6.08% at the animal level and 16.6% at the herd level. The most significant risk factors for *Brucella* seropositivity were mixing of cattle and goats within the same herd and a history of abortions in the herd (*p* < 0.05). This study confirms that caprine brucellosis is endemic in Nyagatare district, highlighting the need for a One Health approach to control and prevent the disease in both livestock and humans. The study recommends implementing awareness campaigns to educate livestock farmers about brucellosis and calls for further research to characterize *Brucella* spp. in small ruminants in Rwanda and to establish appropriate control measures.

## 1. Introduction

Brucellosis is one of the most widespread zoonotic infections globally, caused by *Brucella* species. *Brucella* bacteria are Gram-negative coccobacilli that are nonmotile [1, 2]. Different *Brucella* species affect various hosts: *B. abortus* primarily causes brucellosis in cattle, *B. suis* affects pigs, and *B. melitensis* infects goats and sheep. All these species are pathogenic to humans [2, 3]. In livestock, brucellosis causes sterility and reproductive failure, often leading to abortion and either complete or delayed infertility in affected hosts. Infected animals shed *Brucella* organisms in their milk and through uterine discharges following abortion or parturition [4–7]. Several risk factors influence the spread of brucellosis both within and between herds. A major factor is the introduction of new animals that are asymptomatically infected and not quarantined [8]. Additionally, a history of abortions within a herd significantly increases the risk of disease transmission [9, 10]. The mixing of different breed of herds during grazing or at shared watering places further amplifies the risk of brucellosis transmission [11–13].

Numerous serological tests are employed to screen for *Brucella* infection in livestock. However, no single serological test is universally suitable for all animal species and conditions. Positive screening test results must be confirmed with confirmatory serological tests [2], as serological tests are not fully specific and may cross-react with other bacterial infections, particularly *Yersinia enterocolitica O:9* [14]. The Rose Bengal Test (RBT) is a rapid and straightforward agglutination test that uses an antigen stained with Rose Bengal and buffered to a pH of 3.65 [15]. For small ruminants, the recommended confirmatory serological test is the indirect enzyme-linked immunosorbent assay (iELISA), which uses smooth lipopolysaccharide (sLPS) antigen [16–19]. Although culture remains the gold-standard diagnostic method, it requires specialized expertise and well-equipped laboratory facilities due to associated biohazards. Additionally, its success rate is low because of the fastidious nature of *Brucella* species. While molecular techniques play a significant role in diagnosis, their practical application in field settings is limited. Therefore, there is an urgent need to develop on-site diagnostic approaches with high sensitivity and specificity to ensure accurate detection [20].

In 2018, Rwanda had an estimated 2.1 million agricultural households, with livestock ownership distributed as follows: 61.0% of households owned cattle and 53.6% owned goats [21]. The country's livestock population comprised approximately 2,283,445 goats, 1,856,490 cattle, 703,145 pigs, and 499,316 sheep [21]. The goat population decreased by 6.5%, a decline attributed to high consumption rates and susceptibility to animal diseases [22]. Rwanda has experienced outbreaks of various zoonotic diseases, many of which have become endemic and posing serious threats to both animal and public health. These include brucellosis, Rift Valley fever, rabies, *tuberculosis*, and cysticercosis [23–26]. A study conducted in Rwanda's Huye district, involving women with abortion and stillbirth complications at two hospitals, revealed a 25.0% brucellosis seropositivity rate [27]. At the Rwandan livestock–wildlife–human interface, the seroprevalence of bovine brucellosis was 7.4% at the individual animal level and 28.9% at the herd level [10]. In the Nyagatare district, studies reported bovine brucellosis seroprevalence ranging from 1.7% to 18.9% [28, 29]. In Rwanda, molecular studies detected *Brucella* DNA in 5.6% of slaughtered cattle, identifying both *B. abortus* and *B. melitensis*, with some cases of mixed infections [30]. In the East African Communities (Burundi, Kenya, Rwanda, South Sudan, Tanzania, and Uganda), livestock exhibited an animal-level prevalence of brucellosis ranging from 0.2% to 43.8% in cattle, 0.0%–20.0% in goats, and 0.0%–13.8% in sheep [29]. In humans, the prevalence varied mostly between 0.0% and 35.8% [29]. In Tanzania, *B. abortus*, *B. melitensis*, and undetermined *Brucella* species were identified in dairy cattle, with *B. melitensis* being predominant, despite its typical association with small ruminants [31]. These studies confirmed the endemicity of brucellosis in Rwanda and highlighted human brucellosis as a significant public health threat due to animal contact. However, there has been no research on the prevalence and risk factors of *Brucella* infection in small ruminants in Rwanda [29]. This study therefore aimed to determine the seroprevalence and assess risk factors associated with *Brucella* seropositivity in goats raised in the Nyagatare district of Rwanda. The study hypothesis was that caprine brucellosis is prevalent, with identifiable associated risk factors. The findings of this study will inform future disease control programs and interventions.

## 2. Materials and Methods

### 2.1. Study Area

**Figure 1:**
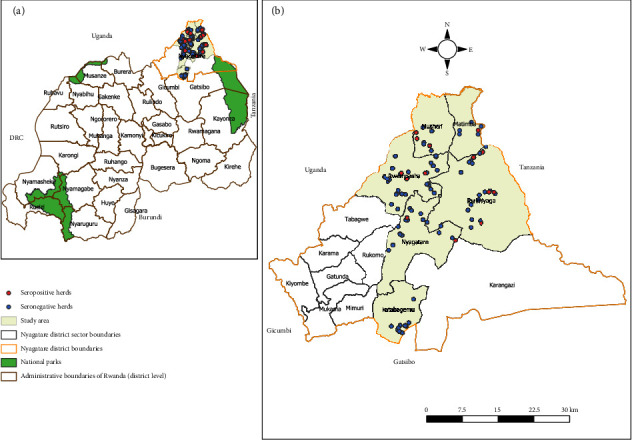
Map of Rwanda (a) with Nyagatare district outlined in yellow. Map of Nyagatare district (b) with sampled sectors (study area) highlighted in green. Red and blue circles show *Brucella* seropositive and seronegative caprine herds found in this study.

The study was conducted in Nyagatare district, one of 30 districts of Rwanda (Figure 1). The district is subdivided into 14 sectors, further divided into 106 cells and 630 villages. The sectors include Nyagatare, Matimba, Rwimiyaga, Rwempasha, Karangazi, Rukomo, Katabagemu, Karama, Kiyombe, Mukama, Tabagwe, Mimuli, Gatunda, and Musheri. Nyagatare district shares borders with Uganda, Tanzania, and Gatsibo districts. It covers an area of 1919 km^2^ and is situated at an altitude of 1414 m. Of the 14 sectors, 6 were selected for sampling based on their high goat populations, as determined by the livestock census data from the Nyagatare District Veterinary Officer. The selected sectors for the study were Nyagatare, Rwempasha, Rwimiyaga, Matimba, Katabagemu and Musheri (Figure 1). Nyagatare district has a population of 465,855 [32]. The average population density is 243 inhabitants per km^2^, which is lower than the national average of 521 inhabitants per km^2^ [32].

### 2.2. Study Animals

Female and male goats older than 6 months with no brucellosis vaccination history were selected. The district veterinary office report from 2017 to 2018 indicated that Nyagatare district has 63,808 goats (District Veterinary Services records). The goat rearing systems in Nyagatare include free ranching, semi-intensive, and tethering. The predominant goat breed in Rwanda is the East African goat (97%), with crossbreds of local and exotic breed (1.7%) [33].

### 2.3. Study Design and Sample Size

A cross-sectional study was conducted from November 2020 to September 2021 in the six sectors of the Nyagatare district. A multistage sampling method was used to sample the herd as the primary sampling unit. The herd sample size was calculated using the formula for simple random sampling suggested by Thrusfield [34]

(1) $$\begin{array}{c} N=\frac{1.96^{2}\times p\left(1-p\right)\;}{d^{2}}. \end{array}$$

The herd sample size was determined using a 95% confidence interval level with an expected herd caprine brucellosis prevalence (*p*) of 13% as previously reported in Uganda [9] and with a desired absolute precision (*d*) of 10%. The formula yielded 44 herds, and the sample size was increased twofold to be 88 to increase the precision [35–37]. Besides, a contingency of 15% of herds was added, leading to a total of 102 herds. The number of herds that were randomly sampled within each sector was based on the proportionate sampling scheme and formed part of the initial stage sampling. The herd sample size in each sector was determined using an Excel random generator.

Within each herd, all goats over 6 months of age were grouped in an enclosed pen and counted. A list of ear tags for all eligible goats (from 1 to *N* where *N* represents the total number of eligible goats in the herd) was created by recording ear tags. Each ear tag was assigned a number from 1 to *N*, then six eligible goats were randomly selected by picking numbers from a hat. Therefore, the total sample size in all 102 herds was 612 goats. As suggested by Dohoo et al. [37], and for financial reasons, to ensure that all animals in the same herd had an approximately equal probability of being selected, herds with fewer than 20 goats or more than 25 goats were excluded from the study. These herds were replaced by the next herd on the randomly generated Excel list that met the herd size criteria. In this study, a herd was defined as all animals reared in the same household during the visit. Seroprevalence data were obtained through laboratory analysis of blood serum samples.

### 2.4. Blood Sample Collection

Blood collection was performed from the jugular vein of goats using nonanticoagulant tubes. A total of 4 mL of blood was drawn from each goat and transported under cold chain conditions in a cooler box at 4°C [38]. The samples were kept at room temperature overnight in the laboratory to allow clotting. Once clotted, the sera were separated and collected into labeled Eppendorf tubes, which were then stored in a freezer at −20°C until analysis. All laboratory analyses were conducted at the University of Rwanda, School of Veterinary Medicine.

### 2.5. Rose Bengal Test (RBT)

Serum samples were analyzed using the RBT with an antigen reagent sourced from ID Vet (France) according to the protocol previously described by Alton et al. [1]. Equal amounts of serum (30 μL) and RBT antigen were placed on a plastic plate. Immediately after the final antigen drop, the serum and antigen were thoroughly mixed to form a round surface with a 2 cm diameter. The mixture was agitated for 4 min at room temperature. After this period, any visible agglutination reaction was recorded as positive. To ensure the reliability and accuracy of the test, positive and negative controls were included in each round of testing, using positive and negative sera provided by the manufacturer.

### 2.6. Indirect Enzyme-Linked Immunosorbent Assays (i-ELISA)

All serum samples were analyzed using an indirect ELISA kit for brucellosis detection (ID Vet, France) following the manufacturer's protocol as described previously by [10, 39]. The iELISA utilizes *Brucella* lipopolysaccharide (LPS) as an antigen to detect anti-*Brucella* antibodies in the serum of small ruminants [40]. Positive and negative controls were included in each test. Both samples and the controls were diluted at 1/20, and if anti-*Brucella* antibodies were present, they formed an antibody–antigen complex. Horseradish peroxidase (HRP) conjugate was added to form an antigen–antibody–HRP complex. After three washes to remove excess conjugate, the substrate TMB (3, 3′, 5, 5′-tetramethylbenzidine) was added, producing a color reaction depending on the quantity of specific antibodies. The color reaction was measured at 450 nm using a Multiskan spectrophotometer (Thermo Scientific, USA). For a valid test, the positive control's mean had to exceed 0.350 (OD_PC_ > 0.350), and the ratio of positive to negative control values had to be greater than 3 (OD_PC_/OD_NC_ > 3). A positive result was recorded when a sample showed an *S*/*P* value greater than or equal to 120%. *S*/*P*% was calculated as: *S*/*P*% = [(OD sample − OD_Nc_)/(OD_Pc_ − OD_Nc_)] × 100 with OD = optic density, Pc = positive control, and Nc = negative control.

### 2.7. Assessment of Risk Factors Associated With Brucellosis

#### 2.7.1. Questionnaire Administration

Information on potential risk factors, including parity, mixing small and large ruminants, contact with other herds, management and breeding practices, introduction of a new goat in the flock, flock size, and history of abortion was collected from the head of the selected herd or household using a structured questionnaire. The questionnaire was designed, tested, and piloted on 10 goat farms to ensure clarity and reliability, and translated into the local language (Kinyarwanda).

#### 2.7.2. Risk Factor Analysis

R Studio and MS Excel 2013 were used for data entry, cleaning, and analysis [41]. The univariable logistic regression analysis was performed to screen for significant associations between the risk factors and the *Brucella* seropositivity. All risk factors with *p*-value ≤ 0.20 in the univariable logistic regression analysis were included in the initial model of the multivariable logistic regression analysis. The model was built using a stepwise selection procedure, where the least significant independent variable was dropped in each step, continuing until all remaining predictor variables were statistically significant (*p*-value ≤ 0.05).

### 2.8. Ethics Approval and Protocol

Assessment and approval of the study protocol were done by the University of Rwanda and the Faculty of Veterinary Science and Faculty of Humanities from the University of Pretoria (ref number: REC032-20). In each administrative cell, goat farmers attended a meeting to discuss the aim of this research. Study participants were not obliged to participate in the study or to allow collection of the blood from their animals. The questionnaire was explained to the participants, and they were encouraged to ask questions for clarification. Participants who provided verbal consent to partake in the questionnaire and allowed blood sampling of animals were enrolled in the study. The farmers' names, regions, and villages were recorded in a nondisclosure agreement. The questionnaire was coded and saved in Excel. For analysis, codes were utilized to substitute farmer names. Blood samples were collected by qualified veterinarians in accordance with animal husbandry regulations.

## 3. Results

### 3.1. Management Practices, Herd Composition, and Farmers' Awareness of Brucellosis

We visited 102 goat's herds distributed across six sectors in the Nyagatare district (Figure 1) for sample collection and simultaneously asked a set of questions to the herd owners. Only 20.6% (21/102 of study participants) were aware of the zoonotic nature of brucellosis. Of the respondents, 95 out of 102 (93.1%) raised their goats alongside other animals (mixed herds). Only 3.9% of respondents kept their goats and other animals separately. The goat herds were mixed with cattle in 87.3% of cases, with dogs in 54.9%, with poultry in 47.1%, and with sheep in 30.4%. Additionally, 82.9% of the respondents indicated that their goats shared pastures and watering points with other animals.

### 3.2. Individual Animal Seroprevalence of Brucellosis

Of the 612 goat samples, 6.8% (42/612) tested seropositive for brucellosis using RBT, while 10.78% (66/612) were seropositive using iELISA. The true seroprevalence was 6.08% for RBT and 10.78% for iELISA. All RBT seropositive samples were also positive using iELISA, indicating that 6.8% of the goats were seropositive by both iELISA and RBT in Nyagatare district. Additionally, 24 samples tested positive with iELISA but negative with RBT. The highest seroprevalence was found in the Rwimiyaga sector with 18.67% followed by Matimba with 5% positive cases by both RBT and i-ELISA (Table 1).

### 3.3. Brucellosis Seroprevalence at Herd Level

The herd was confirmed to be positive when at least one goat in the herd was confirmed to be positive for both RBT and iELISA. The herd-level seroprevalence was 16.6% (17/102) in Nyagatare district (Table 2).

### 3.4. Factors Associated With Brucellosis Herd Seropositivity

#### 3.4.1. Univariable Logistic Regression Analysis for Herd-Level Factors Linked to Brucellosis in Goats

In assessing herd-level factors associated with brucellosis in goat herds, different variables were analyzed using univariable logistic regression to determine their relationship with seropositive herds (Table 3). Three risk factors in this study were statistically linked to herd seropositivity. The majority of respondents (66.6%) reported mixing their cattle and goat herds, which was identified as a significant risk factor. Goat herds mixed with cattle had an eightfold increased risk of testing positive for brucellosis compared to goat herds not mixed with cattle, and this was statistically significant (OR = 8; *p* = 0.05) (Table 3).

Abortions were reported in 68.6% (70/102) of the goat herds. Among the seropositive herds, 94.1% (16/17) had experienced abortion, compared to 63.5% (54/85) of seronegative herds. Herds with a history of abortion had a ninefold increased likelihood of having seropositive animals when compared to herds without such a history, making this association statistically significant (OR = 9.19; *p* = 0.04). However, a history of abortion in other animals was not linked to *Brucella*-positive goat herds (OR = 0.95; *p*-value=0.940) (Table 3).

Interaction with other goat flocks during grazing or watering was observed in 71.5% of the herds. Goat herds that came into contact with other herds during these activities were eight times more likely to test positive for brucellosis compared to herds without such a contact, showing statistical significance (OR = 7.85; *p* = 0.05). However, contact between goat herds and wildlife was not significantly associated with brucellosis seropositivity (OR = 2.0; *p* = 0.320) (Table 3). None of the respondents vaccinated their animals, and the majority (82.4%) did not receive veterinary advice on managing their goats. A small proportion (17.7%) of the respondents reported quarantining new animals before introducing them into the herd.

#### 3.4.2. Multivariable Logistic Regression Analysis Between Potential Risk Factors and *Brucella* Herd Seropositivity

The multivariable logistic regression model identified two risk factors associated with *Brucella* seropositivity in goat herds. Mixing cattle and goat herds was a significant risk factor, with an adjusted odds ratio (aOR = 8.35; *p* = 0.04). Additionally, a history of abortion in the goat herds was independently associated with seropositivity, with an aOR of 0 7.94 (*p* = 0.05). However, contact between goat herds during grazing and watering was not statistically significant (aOR = 6.00; *p* = 0.09) (Table 4).

## 4. Discussion

### 4.1. Seroprevalence of Brucellosis in Goats

This study presents the first documented seroprevalence of caprine brucellosis in Rwanda, with an individual-level true prevalence of 10.7% by iELISA and 6.08% by RBT. Notably, it identified a history of abortion and mixed cattle goat herds as the primary risk factors for herd-level seropositivity. These findings underscore key transmission pathways and highlight the diagnostic limitations of relying exclusively on RBT, which remains the most commonly used test in Rwanda.

All RBT-positive samples in this study were also positive by iELISA; however, 24 samples tested positive with iELISA but negative with RBT. This discrepancy may be explained by differences in the sensitivity and specificity of the tests [42, 43]. The higher detection rate by iELISA may result from its ability to detect a broader range of antibody classes (IgM, IgG1, and IgG2). iELISA can identify antibodies that persist long after peak infection (3–4 weeks post-infection) and may remain detectable for years. In contrast, the IgM response detected by RBT appears early but may wane shortly after infection [44–50]. Additionally, cross-reactivity with *Yersinia enterocolitica* O:9 may explain differences in seropositive results between the two tests [14]. The iELISA cutoff value requires validation in Rwanda, as thresholds developed in low-prevalence regions (e.g., Europe) may underestimate or overestimate local prevalence. Further research is needed to determine whether iELISA-positive but RBT-negative goats have chronic infections, which could be confirmed by bacterial culture. As none of the respondents had vaccinated their goats, positive results are unlikely due to prior immunization. Reliance on RBT alone, as commonly practiced in Rwanda, may therefore be insufficient. Goats testing positive by iELISA but not by RBT may still pose a risk of transmitting brucellosis, especially through animal trade or redistribution programs such as the Rwandan government's initiative to reduce poverty by providing small ruminants to vulnerable households.

The seroprevalence reported in this study for caprine brucellosis in Rwanda was notably lower than the highest seroprevalence reported in cattle within the same district (Nyagatare), which ranged from 14.1% to 18.9% [10, 28]. This difference may be explained by the differing *Brucella* species that primarily infect cattle and goats, as well as by variations in sampling strategies and livestock management practices, particularly the common use of shared bulls among dairy farms, which increases the risk of disease transmission [51]. The seroprevalence observed in this study was lower than that reported in similar studies conducted in Uganda (8.8%), Tanzania (11.49%), and Kenya (13%), all of which used RBT [52–54]. Such variation in seroprevalence across regions may reflect differences in livestock production systems, agroecological zones, study designs, and sample sizes [46, 55–57].

The endemic presence of caprine brucellosis in Nyagatare district, as demonstrated by this study, raises significant public health concerns. *Brucella melitensis*, the species primarily affecting goats, is responsible for most human brucellosis cases, particularly among veterinarians and farmers who handle infected reproductive materials without protection [56, 57]. There is an urgent need for public health education campaigns to raise awareness about brucellosis transmission from goats to humans, particularly targeting practices like handling aborted fetuses without protection. Strengthening collaboration between veterinary and public health sectors using a One Health approach is crucial for effective surveillance and control of brucellosis in both animals and humans. Regular testing, mass vaccination, and culling of seropositive animals may be necessary to control the disease in highly affected areas [20].

### 4.2. Herd-Level Risk Factors Influencing Brucellosis in Goats

This study identified a history of abortion as a significant predictor of *Brucella* infection in goat herds. A recent study in Nyagatare district also isolated *B. melitensis* from goat flocks with recurrent abortions [58]. This finding supports the use of reproductive history as a screening tool in resource-limited settings, where laboratory diagnostics may not be readily available. Abortion in small ruminants is a major clinical sign of *Brucella* infection [9, 59, 60]. Female animals infected with brucellosis release a high concentration of *Brucella* spp. in their milk, placenta, and aborted materials [7]. The release of *Brucella* microorganisms by infected female animals occurs for a long period, which results in contamination of the environment [56, 61]. The spread of brucellosis may be increased by respondents' practices of retaining animals with a history of abortion in the farms, and practices of discarding aborted fetuses in the environment, which may facilitate disease spread within and between herds during grazing or watering. The history of abortion, characterized in this study as the main risk factor, can favor zoonotic transmission of brucellosis through skin abrasions or mucous membranes, as most farmers and animal handlers reported assisting parturition or handling aborted materials without protective clothing [10]. The study indicated a high rate of abortions (68.3%) in goat herds of which 64% were recorded in seronegative herds. These findings align with findings reported by Mourad [62] regarding the high abortion rate of goats in Rwanda. The history of abortions recorded in 64% of seronegative herds may be attributed to various unidentified causes that may include other endemic zoonotic diseases like Rift Valley fever, *Campylobacter fetus*, and *Leptospira spp*. [25, 58]. Implementation of strict biosecurity measures, especially during parturition and abortion events, is essential to reduce disease spread within and between herds and humans.

The association between mixed herds and seropositivity observed in this study is consistent with findings from the study conducted in other neighboring countries Uganda and Tanzania [63, 64], Furthermore, a study conducted in Kenya revealed the transmission of *Brucella abortus* between cattle and goats [65]. These regional findings underscore the importance of species-specific herd management and the need for separation of livestock species to reduce the risk of interspecies transmission. Transmission of *Brucella* spp. between cattle and goat herds could easily be facilitated by improper disposal of aborted fetuses [61, 66] and exacerbated by the absence of a vaccination campaign in Rwanda against brucellosis in small animals [33]. The transmission can also be influenced by the fact that in the mixed farm, goat and cattle are maintained under similar conditions sharing common pasture, shelter, and watering points. The identification of mixed cattle–goat herds as a risk factor highlights the need for improved herd management practices, including the separation of species where possible and vaccination against brucellosis in goats.

Although contact between herds during grazing or watering was significantly associated with seropositivity in univariable analysis, it was not retained as an independent risk factor in multivariable analysis. This suggests that its effect may be mediated through interaction with other variables. The findings on interherd contact among goat herds observed in Rwanda may reflect a poor understanding of brucellosis transmission among goat owners and noncompliance with restricted animal movement policies (zero grazing) recommended by the government to control infectious diseases, including brucellosis. Other contributing factors may include the lack of fences around some of the farms, allowing free movement of goats and other animals between farms [67]. Moreover, the shortage of water in the Nyagatare district forces herds of goats to share communal watering points [67, 68]. These shared communal water points in the Nyagatare district [69] not only enable animal-to-animal transmission of brucellosis but also pose a significant risk for zoonotic transmission to humans, exemplifying the interconnectedness of animal, human, and environmental health central to the One Health approach.

The study revealed that lending breeding males was not significantly associated with herd seropositivity, which contrasts with similar studies conducted in different regions, including neighboring countries, Uganda and Kenya [9, 70]. The lack of association between lending breeding males and seropositivity may reflect informal risk assessments by farmers, who reported choosing males from herds without visible reproductive issues. While studies in Uganda and Kenya identified veterinary services as a protective factor [9, 70], the lack of association observed in our study highlights critical service delivery gaps in the Nyagatare context.

Compared to the 10 well-documented risk factors for bovine brucellosis in Rwanda [10, 28, 71], the current study identified only two for goats, underscoring a significant gap in small ruminant research. The limited data on goats may hinder effective control measures, reinforcing the need for targeted studies and interventions. In Nyagatare district, several conditions including porous borders, communal grazing, and water scarcity may facilitate zoonotic transmission. Cross-border disease control measures may be necessary, given Nyagatare's location and the potential for disease spread from neighboring countries. Implementing a brucellosis vaccination program for goats, particularly in high-risk areas like Nyagatare district, should be considered. Moreover, an effective brucellosis control program should account for multiple factors, including an understanding of regional and local epidemiological variations, cross-sectoral coordination and surveillance, farming practices, available infrastructure, awareness initiatives, and prevailing social customs [20, 72]. The lack of research on brucellosis in species other than cattle and small ruminants poses a significant challenge to the implementation of effective control measures [73].

### 4.3. Study Strengths and Limitations

This study had several notable strengths. It was the first comprehensive investigation of caprine brucellosis in Rwanda, providing crucial baseline data for the Nyagatare district. The robust multistage sampling methodology, covering 102 herds across six sectors, enhanced the representativeness of the findings. The use of both RBT and iELISA for serological testing increased the reliability of prevalence estimates, and the extensive risk factor analysis offered valuable insights for disease control strategies.

However, this study had certain limitations. The geographic scope is restricted to one district, potentially reducing its applicability to the entire country. Additionally, the absence of bacterial isolation or molecular typing to identify specific *Brucella* species and the lack of direct assessment of human brucellosis cases represent important areas for future research. Despite these limitations, this study provides a solid foundation for understanding caprine brucellosis in Rwanda and guides future research and control efforts.

## 5. Conclusions and Recommendations

This study confirmed the endemicity of brucellosis in Nyagatare district, with an adjusted true seroprevalence of 6.08% based on RBT and 10.7% based on iELISA at the individual animal level and 16.6% at the herd level. Multivariable logistic regression analysis identified mixing cattle and goat herds (aOR = 8.35; *p* = 0.04) and a history of abortion (aOR = 7.94; *p* = 0.05) as independent risk factors for brucellosis seropositivity in goat herds. These findings suggest that brucellosis transmission is likely facilitated within mixed herds and across different herds. The identification of these key risk factors emphasizes the need for better herd management and biosecurity measures to mitigate the spread of the disease. The study also highlighted that the majority of the community is unaware of the zoonotic nature of brucellosis and its impact on goats. This lack of awareness, coupled with practices that contribute to the spread of the disease, poses a risk not only to livestock but also to human health. The findings underscore the necessity of adopting a One Health approach to control brucellosis, given the interconnectedness of livestock species and the zoonotic potential of the disease. The study recommends launching awareness campaigns to educate livestock farmers about biosecurity measures, particularly when handling aborted fetuses, and enhancing veterinary services. Future research should focus on characterizing *Brucella* species in small ruminants in Rwanda to gather comprehensive data on the disease and develop targeted control strategies.

## Figures and Tables

**Table 1:** Apparent seroprevalence (AS) and true seroprevalence (TS) of brucellosis among individual goats in different sectors of Nyagatare district using RBT and iELISA.

Sector	Goats tested	RBT + *n* (%)	RBT AS (95% CI)	RBT TS (95% CI)	iELISA + *n* (%)	iELISA AS (95% CI)	iELISA TS (95% CI)
Rwimiyaga	166	31 (18.7)	18.67 (12.75–24.6)	17.9 (12.02–23.97)	43 (25.9)	25.9 (19.24–32.57)	25.9 (19.24–32.57)
Rwempasha	207	7 (3.4)	3.38 (0.92–5.84)	2.57 (0.09–5.06)	12 (5.8)	5.8 (2.61–8.98)	5.8 (2.61–8.98)
Musheli	44	1 (2.3)	2.27 (−2.13–6.68)	1.45 (−2.99–5.9)	4 (9.1)	9.09 (0.6–17.59)	9.09 (0.6–17.59)
Nyagatare	82	1 (1.2)	1.22 (−1.16–3.6)	0.39 (-2–2.79)	3 (3.7)	3.66 (−0.4–7.72)	3.66 (−0.4–7.72)
Matimba	40	2 (5.0)	5.00 (−1.75–11.75)	4.2 (−2.61–11.02)	4 (10.0)	10.00 (0.7–19.3)	10.00 (0.7–19.3)
Katabagemu	73	0 (0.0)	0.00 (0–0)	0.00 (−0.84–0.84)	0 (0.0)	0.00 (0–0)	0.00 (0–0)
Total	612	42 (6.9)	6.86 (4.86–8.87)	6.08 (4.06–8.10)	66 (10.8)	10.78 (8.33–13.24)	10.78 (8.33–13.24)

Abbreviations: AS, apparent seroprevalence; CI, confidence interval; iELISA, indirect enzyme-linked immunosorbent assay; RBT, Rose Bengal Test; TS, true seroprevalence.

**Table 2:** Number of herds with brucellosis positive goats in Nyagatare district.

The number of goats seropositive in the flock	Number of herds with positive reactors on both RBT and iELISA	Number of herds with positive reactors using iELISA
1	6	8
2	4	5
3	1	2
4	4	5
5	2	3
6	0	1
Total	17	24

**Table 3:** Univariable logistic regression analysis for herd-level factors linked to brucellosis in goats.

Variables	Categories	Number of herds visited	Herd positive *n* (%)	Odds ratio (95%-CI)	*p* value
Mixing cattle and goat herds	No	27	1 (3.7)	8 (1.50–148.30)	0.05
	Yes	68	16 (23.5)		

Raising animals together or separately	Raise animals together	91	16 (17.6)	0.64 (0.08–13.39)	0.71
	Raise animals separately	4	1 (25)		

Goat ownership	No	27	5 (18.5)	0.84 (0.28–2.88)	0.76
	Yes	75	12 (16)		

Presence of a calving pen	No	39	8 (20.5)	0.65 (0.22–1.88)	0.41
	Yes	63	9 (14.3)		

Contact with other animal herds during grazing or watering in a year	No	29	1 (3.4)	7.85 (1.48–145.41)	0.05
	Yes	73	16 (21.9)		

Contact with wildlife animals in the past year	No	91	14 (15.4)	2.06 (0.41–8.17)	0.33
	Yes	11	3 (27.3)		

Abortions or stillbirths in the goat herd in the past year	No	32	1 (3.1)	9.19 (1.74–169.75)	0.04
	Yes	70	16 (22.9)		

Abortion of other animals from the herd in the past year	No	72	13 (18.1)	0.96 (0.25–3.08)	0.94
	Yes	23	4 (17.4)		

Introduction of a new goat in the herd in the past 12 months	No	49	9 (18.4)	0.79 (0.27–2.26)	0.66
	Yes	53	8 (15.1)		

Borrowing male animals for breeding to other herds in the past year	No	23	3 (13)	1.44 (0.42–6.68)	0.6
	Yes	79	14 (17.7)		

Received veterinary extension services on the management of goat herds	No	84	14 (16.7)	1 (0.21–3.56)	1
	Yes	18	3 (16.70)		

Abbreviations: CI, confidence interval; OR, odds ratio.

**Table 4:** Multivariable logistic regression model of factors associated with herd-level brucellosis seropositivity in goats.

Sn	Variables	Odds ratio	95% CI	Coefficient	S.E	*Z*-statistic	*p* value
1	Mixing of cattle and goat herds	8.35	1.48–157.66	2.122	1.076	1.97	0.04
2	Abortion history in goat herd.	7.94	1.40–150.16	2.072	1.078	1.92	0.05
3	Contact between goat herds	6.09	1.05–116.03	1.807	1.085	1.66	0.09

Abbreviations: CI, confidence interval; OR, odds ratio; SE, standard error.

## Data Availability

The data that support the findings of this study are available on request from the corresponding author. The data are not publicly available due to privacy or ethical restrictions.
